# Grain Refinement Kinetics in a Low Alloyed Cu–Cr–Zr Alloy Subjected to Large Strain Deformation

**DOI:** 10.3390/ma10121394

**Published:** 2017-12-06

**Authors:** Anna Morozova, Elijah Borodin, Vladimir Bratov, Sergey Zherebtsov, Andrey Belyakov, Rustam Kaibyshev

**Affiliations:** 1 Laboratory of Mechanical Properties of Nanostructured Materials and Superalloys, Belgorod State University, Belgorod 308015, Russia; belyakov@bsu.edu.ru (A.B.); rustam_kaibyshev@bsu.edu.ru (R.K.); 2 Institute of Problems of Mechanical Engineering RAS, Saint-Petersburg 199178, Russia; elbor7@gmail.com (E.B.); vladimir.bratov@gmail.com (V.B.); 3 Department of Elasticity, Saint-Petersburg State University, Saint-Petersburg 199034, Russia; 4 Laboratory of Bulk Nanostructured Materials, Belgorod State University, Belgorod 308015, Russia; zherebtsov@bsu.edu.ru

**Keywords:** Cu–Cr–Zr alloy, grain refinement, severe plastic deformation, triple junctions, grain refinement kinetics

## Abstract

This paper investigates the microstructural evolution and grain refinement kinetics of a solution-treated Cu–0.1Cr–0.06Zr alloy during equal channel angular pressing (ECAP) at a temperature of 673 K via route B_C_. The microstructural change during plastic deformation was accompanied by the formation of the microband and an increase in the misorientations of strain-induced subboundaries. We argue that continuous dynamic recrystallization refined the initially coarse grains, and discuss the dynamic recrystallization kinetics in terms of grain/subgrain boundary triple junction evolution. A modified Johnson–Mehl–Avrami–Kolmogorov relationship with a strain exponent of about 1.49 is used to express the strain dependence of the triple junctions of high-angle boundaries. Severe plastic deformation by ECAP led to substantial strengthening of the Cu–0.1Cr–0.06Zr alloy. The yield strength increased from 60 MPa in the initial state to 445 MPa after a total strain level of 12.

## 1. Introduction

The Cu–Cr–Zr alloys are one of the typical Cu-base precipitation hardening-type alloys, which were designed to exhibit both high electrical conductivity and high strength [1,2,3,4,5,6,7,8,9,10,11]. These alloys are advanced materials for railway contact wire, resistance welding electrodes, electronic commutators, etc. [2,12]. There is a demand for high-speed electric railways to increase the strength and electroconductivity of contact wires [4,13]. The strength of Cu–Cr–Zr alloys can be significantly enhanced through grain refinement in accordance with the Hall–Petch equation [14,15,16,17,18,19,20,21]. One of the promising methods for grain refinement and the hardening of various metallic materials, including copper and Cu–Cr–Zr alloys, is severe plastic deformation [15,18,19,20,21,22,23,24,25,26,27,28,29,30,31,32,33]. Recently, many techniques of severe plastic deformation such as high pressure torsion [6,22,23], multidirectional forging [24,25], accumulative roll-bonding [26], and equal channel angular pressing [4,18,19,20,21,27,28,29,30,31,32] have been developed to obtain an ultrafine grained structure in metals and alloys. Most of this specific technique is used only for laboratory simulations at the present time. The main advantage of equal channel angular pressing (ECAP) is the possibility of its industrial application as ECAP–Conform to process large semiproducts [21,22,23]. The ECAP–Conform processing can be used in continuous lines of rod or wire production [33,34,35,36]. The microstructures and mechanical properties developed after ECAP and ECAP–Conform have been confirmed as the same [33]. The necessary properties, such as strength and electroconductivity, can be obtained by controlling the development of the ultrafine-grained structure during large strain plastic deformation. 

The development of ultrafine-grained structures in copper and its alloys during severe plastic deformation results from a type of continuous dynamic recrystallization in which the grain refinement can be considered an evolution of the deformation of substructures [19,20,21,37,38,39,40]. During continuous dynamic recrystallization, the strain-induced low-angle boundaries gradually transform into high-angle boundaries with an increase in the total strain. As a result, the ultrafine-grained structure with high dislocation density evolves at large strain levels [19,20,21,37,38,39,40,41]. 

It is important in the dynamic recrystallization that new low-angle boundaries form and increase their misorientations during deformation. The continuous dynamic recrystallization development can be characterized by: the mean angle of boundary misorientations, the fraction of low-angle and high-angle boundaries, the distribution of boundary misorientations, and the nature and distribution of boundary triple junctions formed by the new strain-induced (sub)boundaries. However, the triple junction analysis has not yet been reported in scientific literature. The model distribution of the triple junctions, including special and ordinary grain boundaries, has been briefly discussed [42]. In general, triple junctions in deformed materials can be formed by: three low-angle boundaries (J0), one high-angle and two low-angle boundaries (J1), two high-angle and one low-angle boundary (J2), and three high-angle boundaries (J3). The developing microstructure should correspond to the specific triple junction distribution. Thus, at relativity small strain levels, a large fraction of J0 and a small fraction of J3 are expected. On the other hand, the J3 fraction in the dynamically recrystallized ultrafine-grained structure after large strain deformations should be approximately 1. Therefore, the study of the triple junction evolution can be used as a new approach to follow the microstructural changes and the kinetics of the grain refinement process.

The progress in discontinuous dynamic recrystallization complies with normal Avrami kinetics, and the recrystallized fraction (F_DRX_) can be related to a strain (ε) through a modified Johnson–Mehl–Avrami–Kolmogorov equation [43,44,45],

F_DRX_ = 1 − exp(−k ε^n^), (1)

where k and n are constants, which depend on the material nature and processing conditions. 

The development of the ultrafine-grained structure during cold-to-warm deformation has been studied in numerous papers [20,46,47]. It was shown that the modified Johnson–Mehl–Avrami–Kolmogorov equation could adequately describe the kinetics of continuous dynamic recrystallization. This approach used the ultrafine-grain fraction for the quantitative assessment of the continuous dynamic recrystallization progress. On the other hand, the evolution of triple junctions in dynamic recrystallized microstructures has not been detailed, although it can also be used to characterize the grain refinement kinetics during cold-to-warm deformation. Thus, the aims of the present work are to study the effect of ECAP on the microstructure evolution and the grain refinement kinetics in a Cu–Cr–Zr alloy using the triple junction distribution analysis.

## 2. Materials and Methods

A Сu–Cr–Zr alloy (Cu–0.1Cr–0.06Zr, all in wt %) subjected to a solution treatment at 1093 K for 1 h followed by water quenching was used as the starting material. The initial grain size was about 120 μm. ECAP was used as the method of severe plastic deformation. The billets of 14 mm × 14 mm × 90 mm were processed by ECAP via route B_C_ (90° anticlockwise rotation of the samples after each ECAP pass) at a strain rate of 1 s^−1^. The true strain attained at each pass was 1. ECAP processing was executed to different total strain levels, up to 12. The fine microstructure of ECAP samples was examined by a Quanta 250 Nova scanning electron microscope (FEI, Hillsboro, OR, USA) equipped with an electron backscattering diffraction (EBSD) analyzer (FEI, Hillsboro, OR, USA) using an orientation imaging microscopy (OIM) software (OIM Analysis 5.2.0, EDAX TSL, Mahwah, NJ, USA). The microstructural investigations were carried out on the Y plane, i.e., flow plane along the side face at the point of exit from the die [27]. The specimens for the EBSD analysis were electrochemically polished at 238 K using an electrolyte of HNO_3_:CH_3_OH = 1:3. The step size for the EBSD scan was t = 420 nm for the specimen deformed to a total strain of ε = 1, t = 200 nm for the specimen deformed to ε = 2, and t = 50 nm for specimens deformed to total strain levels of four to 12. The OIM images were processed by the clean-up procedures, setting a minimal confidence index of 0.1. The mean grain size (D) was measured by the linear intercept method on the OIM images as an interval between high-angle boundaries. A critical misorientation angle between low-angle and high-angle boundaries was 15°. The dislocation densities were estimated using the kernel average misorientations over a distance of 400 nm [21]. The fraction of high-angle boundaries (F_HAB_) and ultrafine grains (F_UFG_), i.e., those with D < 2 μm, were evaluated using the OIM software. The triple junctions fraction was estimated counting more than 300 junctions for each state. The tensile tests were executed at ambient temperature using an Instron 5882 (Illinois Tool Works Inc., Norwood, MA, USA) tensile machine with an initial strain rate of 2 × 10^−3^ s^−1^. 

## 3. Results

### 3.1. Microstructural Evolution

**Figure 1:**
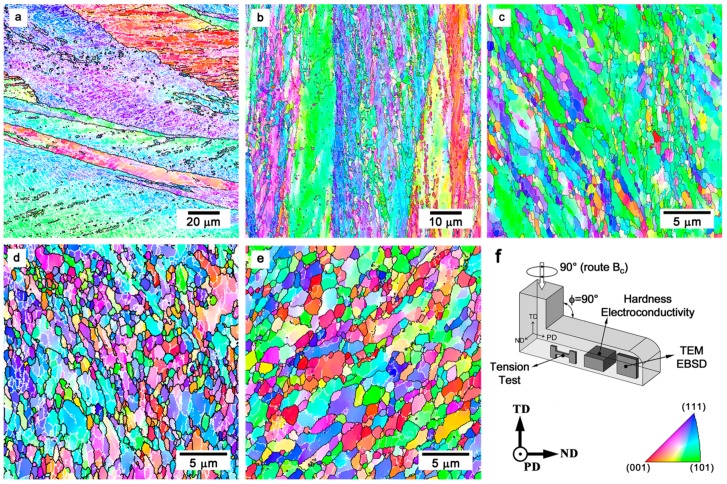
Typical deformation microstructures developed in a Cu–0.1Cr–0.06Zr alloy subjected to equal channel angular pressing (ECAP) at a temperature of 673 K to total strains of 1 (**a**); 2 (**b**); 4 (**c**); 8 (**d**); and 12 (**e**). The inverse pole figures are shown for the pressing direction (PD) in (**f**). The white and black lines indicate the low-angle (θ < 15°) and high-angle (θ ≥ 15°) boundaries, respectively.

After solution treatment, the initial microstructure of the Cu–0.1Cr–0.06Zr alloy included coarse grains with the size of about 120 μm. The typical deformation microstructures that developed in the Cu–0.1Cr–0.06Zr alloy subjected to ECAP deformation to various strain levels are shown in Figure 1. 

When ECAP was performed on a relativity small strain level of about one, the initial coarse grains elongated along the metal flow direction. The deformation microbands bounded by high-angle boundaries, and many strain-induced subboundaries with low-angle misorientations (θ < 15°), formed within the initial grains. Further deformation resulted in the development of deformation microbands. The deformation microbands separated the initial grains into fragments with a size less than 10 μm. Increasing the transverse boundaries misorientation angle within the deformation microbands with straining resulted in the ultrafine-grain formation. An increase in the deformation microband number promoted the development of new ultrafine grains, and lead to the partially recrystallized microstructure. After a total strain level of eight, many subboundaries transformed into high-angle boundaries, and uniform equiaxed grains with a mean grain size below 1 μm developed. Further deformation did not lead to any qualitative changes in the deformation microstructures. 

**Figure 2:**
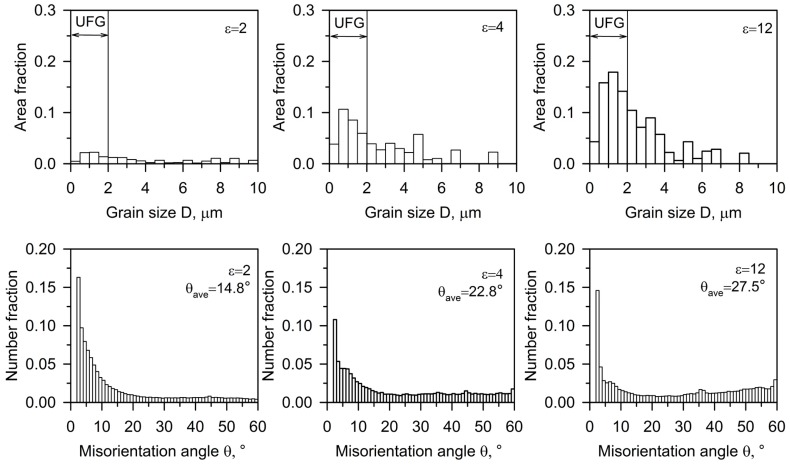
Grain size and boundary misorientation distributions for a Cu–0.1Cr–0.06Zr alloy processed by ECAP at 673 K to total strains (ε) of two to 12.

As can be seen in Figure 2, the grain size distribution is characterized by a low fraction of the ultrafine-grained structure near 0.05 at a relatively small strain of ε ~ 2. The increasing strain promotes the ultrafine-grain formation and a substantial increase in the ultrafine-grain area fraction after four ECAP passes. The fraction of large grains decreases, while that of fine grains increases upon further processing. Thus, after a total strain level of eight, the ultrafine-grain fraction is above 0.5. Finally, a rather large peak stands out for small grain sizes. 

The (sub)grain misorientations developed in the Cu–Cr–Zr alloy during the severe plastic deformation by ECAP are presented in Figure 2. The large fraction of low-angle boundaries is observed after the second ECAP pass. Then, the maximum against low-angle misorientations gradually decreased, and the fraction of high-angle misorientations increased with straining. Such flat-type misorientation distribution is often observed in various metals and alloys during severe plastic deformation accompanied by continuous dynamic recrystallization, irrespective of the processing method [19,20,21,37,38,39]. 

**Figure 3:**
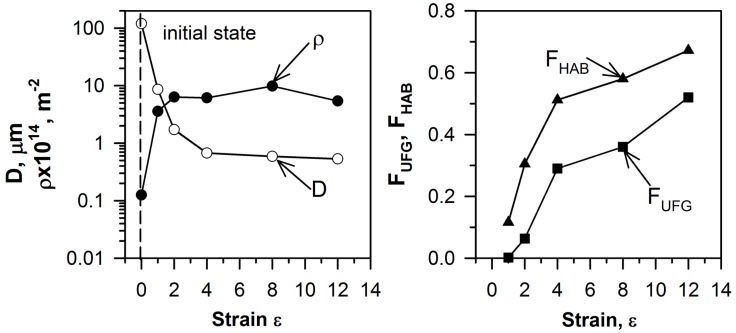
The strain (ε) effect on the mean grain size (D), the dislocation density (ρ), the fraction of high-angle boundaries (F_HAB_), and the fraction of ultrafine grain (F_UFG_) in a Cu–0.1Cr–0.06Zr alloy subjected to ECAP at 673 K.

The changes in the grain size (D), the dislocation density (ρ), the high-angle boundaries fraction (F_HAB_), and the ultrafine-grain fraction (F_UFG_) during ECAP are shown in Figure 3. ECAP produces substantial grain refinement in the range of strain levels from one to four. After the first ECAP pass, the mean grain size drastically reduced to 8.6 μm. Further deformation promoted grain refinement, and the mean grain size after four ECAP passes was less than 1 μm. Then, the rate of grain refinement slowed down; after a total strain of ε = 12, the mean grain size attained 0.5 μm. The ECAP processing was accompanied by a significant increase in the dislocation densities, from 5 × 10^12^ m^−2^ in the initial state to about 9 × 10^14^ m^−2^ after straining to eight. It is seen in Figure 3 that the dislocation density change during ECAP clearly correlates with the reduction in grain size. 

The kinetics of the dynamic recrystallization during large plastic deformation can be estimated using the high-angle boundary fractions (F_HAB_) and ultra-fine grain fractions (F_UFG_). An increase of the ultra-fine grain fraction has an incubation period corresponding to relativity low strains of zero to two. Then, the ultra-fine grain fraction significantly increased, and after a total strain of 12, attained 0.5. In contrast, the high-angle boundaries fraction gradually increased from 0.1 to its apparent saturation of about 0.7, increasing the total strain from one to 12. This behavior of ultrafine grain and high-angle boundary evolution is associated with the microbands, which are bounded by high-angle boundaries, but do not involve ultrafine grains at relatively small strain levels.

### 3.2. Tension Behavior

**Figure 4:**
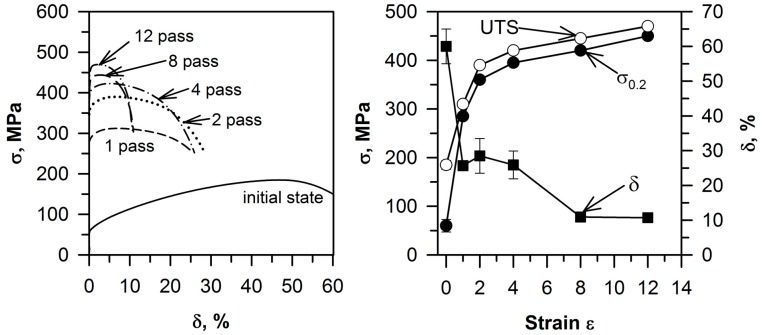
Stress-strain curves and the strain effect on the yield strength, (σ_0.2_), the ultimate tensile strength (UTS), and total elongation (δ) of a Cu–0.1Cr–0.06Zr alloy subjected to ECAP at 673 K.

The solution-treated samples of the Cu–0.1Cr–0.06Zr alloy are characterized by the small yield strength (σ_0.2_) of 60 MPa and the ultimate tension stress (UTS) of 185 MPa, which is comparable to pure copper [16]. The hardening stage is large, and the elongation amounts to 60% in tensile tests (Figure 4). The strain imposed by ECAP to the copper alloy strongly influences its strength and ductility. The first pass results in significant strengthening; σ_0.2_ and UTS increase by about 375% and 70%, respectively. Then, efficiency of deformation strengthening degrades; after the second ECAP pass, additional increments in the both σ_0.2_ and UTS are 75 MPa. Upon further straining (4 to 12 passes), the σ_0.2_ and UTS values increase slowly, leading to gradual strengthening. 

The maximum σ_0.2_ and UTS are 445 MPa and 465 MPa after 12 ECAP passes, respectively. The strengthening by deformation to strain levels of 12 leads to a degradation in the plasticity. Total elongation decreases from 60% in the initial state to 11% after 12 passes of ECAP. The severe plastic deformation of the Cu–Cr–Zr alloy shortens the hardening stage. In contrast to the initial state, the necking in the ECAP processed samples takes place at relatively small tensile strain levels, leading to rapid fracture during the tensile tests. As a result, the UTS and σ_0.2_ values are very close to each other in the Cu–0.1Cr–0.06Zr alloy subjected to the ECAP processing.

## 4. Discussion

The severe plastic deformation is accompanied by significant microstructure change that is associated with an increase in the dislocation density and the strain-induced (sub)boundaries. The new grains develop heterogeneously, that is, assisted with the formation of deformation bands. This process promotes the fragmentation of the initial grains, and leads to a rapid increase in the F_HAB_ fraction, while F_UFG_ does not increase remarkably at early stage of deformation (Figure 3). The number of the deformation microbands rapidly increases during ECAP to a strain level of two. Then, the new ultrafine grains readily develop along the microbands and the initial grain boundaries, as well as at their intersections, accelerating an increase in F_UFG_. The deformation microbands and the new (sub)boundaries lead to the appearance of new triple junctions formed by low-angle and/or high-angle boundaries. The number of high-angle boundaries in the triple junctions and the distribution of the triple junction fractions are controlled by continuous dynamic recrystallization and grain refinement.

**Figure 5:**
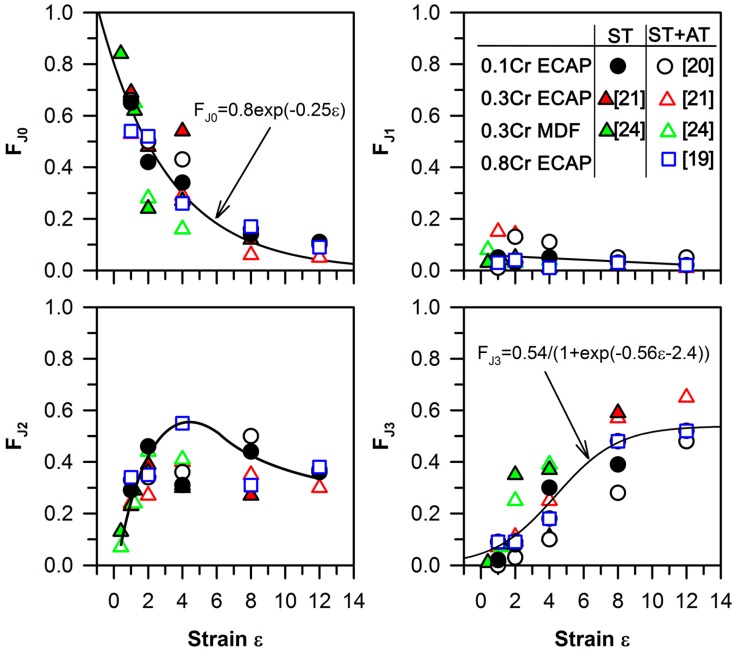
The strain effect on the fraction of triple junctions with zero, one, two, or three adjacent high-angle boundaries, denoted as F_J0_, F_J1_, F_J2_, and F_J3_, respectively, for a Cu–0.1Cr–0.06Zr (0.1Cr, circle [20]), Cu–0.3Cr–0.5Zr (0.3Cr, triangles [21,24]), Cu–0.8Cr–0.05Zr (0.8Cr, square [19]) alloys after solution treatment (ST) or aging (AT) subjected to ECAP or multidirectional forging (MDF) at 673 K.

The effect of total strain on the fraction of triple junctions with the different contents of high-angle boundaries in the present alloy and several other Cu–Cr–Zr alloys with different chromium/zirconium contents subjected to severe plastic deformations by different methods at a temperature of 627 K [19,20,21,24] is represented in Figure 5. The large fraction of J0 at the relativity small strain levels corresponds to the formation of many dislocation subboundaries with low-angle misorientations. Then, the fraction of J0 gradually decreases with straining as the new high-angle boundaries develop. It should be noted that the number of the J1 junctions is quite small, and does not vary remarkably during the deformation, irrespective of total strains. The J1 fraction is almost unchanged with straining, and equals 0.1–0.15. In contrast, many triple junctions with two high-angle boundaries rapidly appear upon the plastic deformation to strain levels of four. Therefore, the J2 fraction quickly increases in the strain range of zero to four, followed by a slight decrease during subsequent deformation. The fraction of J3 exhibits an accelerated increase in the intermediate strain level range of two to eight, and then approaches an apparent saturation of about 0.5 at large strain levels. The strain level range of zero to four is characterized by the development of the deformation microbands. The formation of such bands leads to an increase in the J2 fraction and a decrease in the J0 fraction. The change in the J0 fraction during large deformation of the Cu–Cr–Zr alloys with different Cr and Zr content, as shown in Figure 5, can be related to the strain (ε) through an exponential function:

F_J0_ = 0.8 exp(−0.25 ε).

The high J2 and low J0 fractions indicate the localization of deformation in the microbands. The strain dependence of the J2 fraction on ECAP deformation has a peak at a total strain level of four to six. Further plastic deformation is accompanied by a decrease in the fraction of low-angle boundaries; thus, the J0 fraction degrades to almost zero at sufficiently large strain levels. The transformation of the transverse low-angle boundaries into high-angle boundaries and the ultra-fine grained structure formation lead to an increase in the J3 fraction, while the J2 fraction decreases when the total strains are in the range of 8–12. The strain effect on the J3 fraction in the various Cu–Cr–Zr alloys is shown in Figure 5, and can be approximated by a sigmoid law as follows:

F_J3_ = 0.54/(1 + exp(−0.56 ε − 2.4)).

The faster structural changes during multidirectional forging compared with ECAP may be caused by a frequent rotation of the samples around three orthogonal axes with respect to the forging direction (i.e., in each pass strain of 0.4 during multidirectional forging against a pass strain of 1.0 during ECAP). 

**Figure 6:**
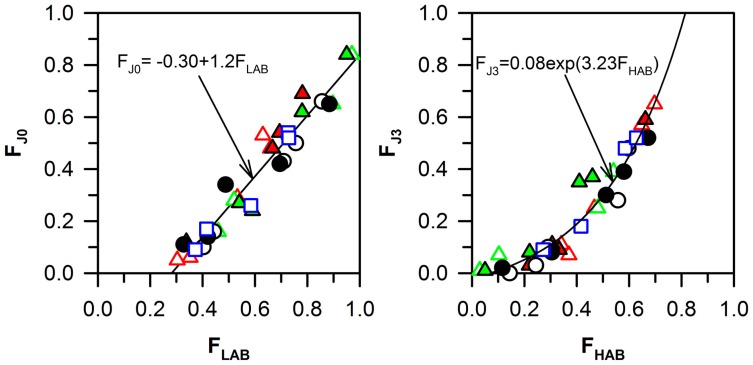
The relationship between the low-angle (F_LAB_) and high-angle (F_HAB_) boundary fractions and the fractions of triple junctions with zero (F_J0_) and three (F_J3_) adjacent high-angle boundaries in the Cu–0.1Cr–0.06Zr (0.1Cr, circle [20]), Cu–0.3Cr–0.5Zr (0.3Cr, triangles [21,24]), Cu–0.8Cr–0.05Zr (0.8Cr, square [19]) alloys after solution treatment (ST) or aging (AT) subjected to ECAP or multidirectional forging (MDF) at 673 K.

The J0 fraction depends on the quantity of low-angle boundaries, and should correlate with the low-angle boundaries fraction. Figure 6 illustrates the relationship between the J0 fraction, including only low-angle boundaries, and the low-angle boundaries fraction. It can be seen that the experimental data can be expressed by a linear function as follows:

F_J0_ = −0.30 + 1.2 F_LAB_

On the other hand, the high-angle boundaries fraction should correlate with the fraction of the J3 triple junctions. The relationship between the change in the J3 fraction and the change in the high-angle boundaries fraction (as seen in Figure 6) can be expressed by an exponential law:

F_J3_ = 0.08 exp(3.23 F_HAB_)

**Figure 7:**
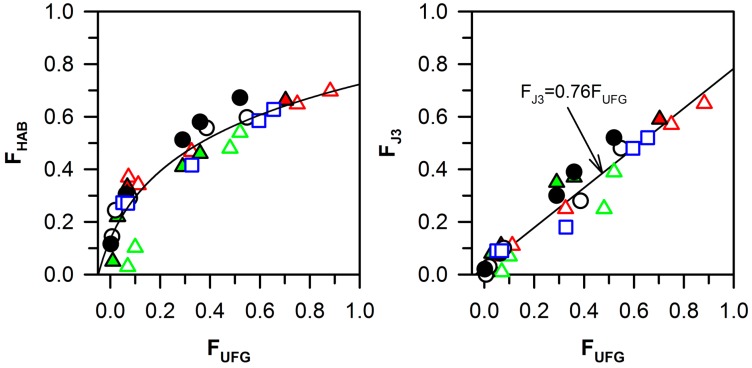
Relationships between the ultrafine-grain fraction (F_UFG_), the high-angle boundary fraction (F_HAB_), and the fraction of the triple junctions of high-angle boundaries (F_J3_) in the Cu–0.1Cr–0.06Zr (0.1Cr, circle [20]), Cu–0.3Cr–0.5Zr (0.3Cr, triangles [21,24]), Cu–0.8Cr–0.05Zr (0.8Cr, square [19]) alloys after solution treatment (ST) or aging (AT) subjected to ECAP or multidirectional forging (MDF) at 673 K.

The high-angle boundaries fraction has been suggested to correlate with the ultrafine-grain fraction [19,20,24,38]. The relationship between F_HAB_, F_UFG_, and F_J3_ is represented in Figure 7. The rapid growth of the high-angle boundaries fraction is associated with the appearance of deformation bands. The ultrafine-grain formation requires the high-angle misorientations for all of the boundaries surrounding the crystallite. Therefore, the formation of ultrafine-grains is delayed at the early deformation stage until the density of high-angle boundaries attains a sufficiently large value. In contrast, the J3 fraction clearly correlates with the ultrafine-grain fraction, and can be expressed by a linear function passing through the origin:

F_J3_ = 0.76 F_UFG_

The triple junctions that consist of only high-angle boundaries and ultrafine-grained structures start to form at the same time, but the rate of ultrafine-grain formation is faster than the increase of the J3 fraction. The lag of the J3 fraction increase is associated with a difference between the upper level of ultrafine-grain size (2 μm) and the subgrain size (0.3–0.5 μm).

The correlation of the J3 fraction with the ultrafine-grain fraction makes it possible to use the J3 analysis to estimate the kinetics of grain refinement that can be discussed in terms of the dynamic recrystallization kinetics using Equation (1). During dynamic recrystallization, the change of the J3 fraction should correspond to the change in the fraction of the dynamic recrystallized grains. Therefore, the Johnson–Mehl–Avrami–Kolmogorov equation using the triple junctions approach has a similar form:

F_J3_ = 1 − exp(−k ε^n^). (2)

**Figure 8:**
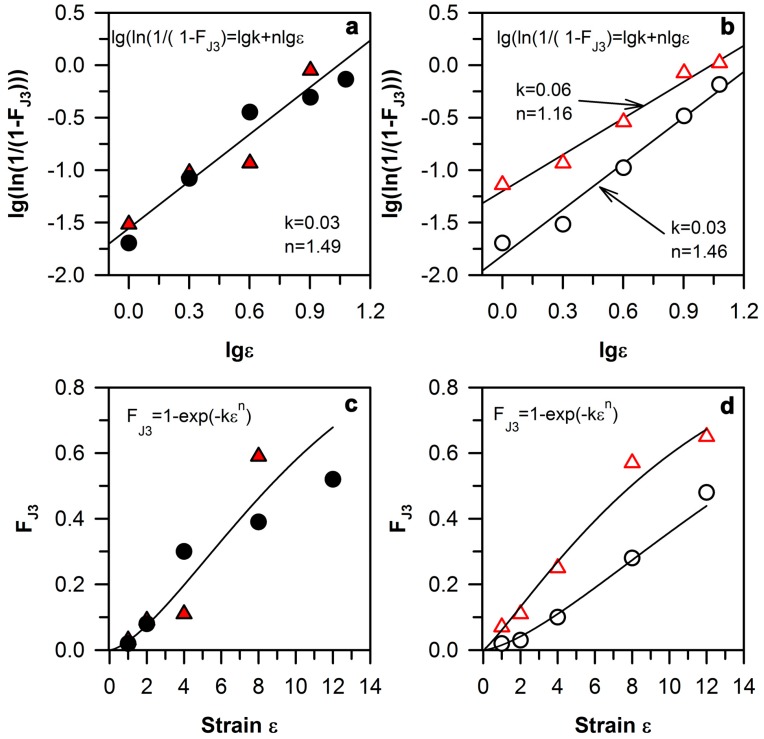
The strain effect on the grain refinement in a Cu–0.1Cr–0.06Zr (circle [20]) and Cu–0.3Cr–0.5Zr (triangle [21]) alloys during ECAP at 673 K; recrystallization kinetics for solution-treated (**a**) and aged (**b**) samples, and the strain effect on the ultrafine-grain fraction in solution-treated (**c**) and aged (**d**) samples.

The plot of ln(1/(1 − F_DRX_)) vs. ε in logarithmic scale should represent a straight line. The change in the J3 fraction for the Сu–0.1Cr–0.05Zr and Cu–0.3Cr–0.5Zr alloys in solution-treated and aged conditions are presented in Figure 8a,b. 

It is clearly seen that the J3 evolution kinetics in these two alloys are nearly the same for the solid solution conditions, and can be described by the Johnson–Mehl–Avrami–Kolmogorov equation with constants of n = 1.49, k = 0.03. On the other hand, the aged Cu–Cr–Zr alloys with different chromium contents demonstrate the different changing rates of the J3 fraction. The chromium increase leads to the acceleration of J3 changing kinetics, constants of n = 1.16, k = 0.06 are obtained for the Cu–0.3Cr–0.5Zr alloy, and those of n = 1.46, k = 0.03 are obtained for the Сu–0.1Cr–0.05Zr alloy. The promotion of the DRX kinetics has been discussed as a result of particle precipitation in the starting materials [48,49]. Therefore, the difference in the volume fraction of Cr and Zr particles in the Cu–Cr–Zr alloys can lead to increasing the J3 changing kinetics in the Cu–0.3Cr–0.5Zr alloy. 

The obtained relationships between J0 and low-angle boundaries, and J3 and high-angle boundaries/ultrafine grains, as well as the clear correlation between the qualitative microstructure evolution and the quantitative variation of the J0, J1, J2, and J3 fractions makes it possible to use the triple junction analysis as an informative source for understanding the structural changes during deformation. The presented approach using the triple junction analysis describes the microstructural evolution during plastic deformation well, and allows us to study the grain refinement and dynamic recrystallization in more detail.

## 5. Conclusions

The grain refinement, microstructural evolution, and kinetics of dynamic recrystallization in the Cu–0.1Cr–0.06Zr alloy subjected to ECAP processing at 673 K were studied using the boundary triple junction analysis. The main results can be summarized as follows:

1. The ECAP processing was accompanied by a significant decrease in the grain size, from 120 μm in the initial condition to 0.5 μm after a total strain of 12. The grain size rapidly decreased during the first four ECAP passes, and then remained almost unchanged during further ECAP.
2. The formation of the ultrafine-grained structure resulted from the deformation of band evolution and an increase in the misorienations of strain-induced subboundaries during ECAP processing. An increase in total strain led to an increase in both the high-angle boundary fraction and the ultrafine-grain fraction. The grain refinement can be discussed in the terms of continuous dynamic recrystallization.
3. The ECAP deformation was accompanied by gradual strengthening. The yield strength increased from 60 MPa in the initial state to 445 MPa after 12 ECAP passes. Correspondingly, total elongation decreased from 60% to 9%.
4. The fraction of boundary triple junctions consisting of only low-angle boundaries gradually decreased through an exponential law function of total strain during severe plastic deformation. The fraction of boundary triple junctions, with one high-angle boundary and two low-angle boundaries was about 0.1–0.15, and did not change remarkably with straining. The fraction of boundary triple junctions with two high-angle boundaries and one low-angle boundary increased to a peak after four to six strain levels, followed by a small decrease at large strain levels. The fraction of boundary triple junctions that consisted of only high-angle boundaries increased by a sigmoid law function with deformation.
5. The fractions of the low-angle boundary triple junctions and the high-angle boundary triple junctions can be related to the low-angle boundary fraction and the ultrafine-grain fraction, respectively, through linear functions. The strain dependence of the high-angle boundary triple junctions can be expressed by a modified Johnson–Mehl–Avrami–Kolmogorov equation, F_J3_ = 1 − exp(−k ε^n^), with a strain exponent of n = 1.49 and k = 0.03.
